# A Case of Recurrent Chromoblastomycosis Treated with Multiple Surgical Management Options

**DOI:** 10.3390/dermatopathology13010010

**Published:** 2026-03-18

**Authors:** Madeleine Kelly, Crystal Williams, Robert Miller

**Affiliations:** 1 Dermatology Department, Townsville University Hospital, 100Angus Smith Drive, Townsville, QLD 4814, Australia; 2 College of Medicine and Dentistry, James Cook University, 1 James Cook Drive, Douglas, Townsville, QLD 4814, Australia

**Keywords:** fungal, surgical management, chromoblastomycosis

## Abstract

Chromoblastomycosis is a chronic mycosis of the skin and subcutaneous tissue typically caused by traumatic inoculation of dematiaceous fungi of the *Herpotrichiellaceae*. A 59-year-old male presented with a 12-month history of an asymmetrical, scaly plaque on the left forearm that has been slowly increasing in size. Past medical history included atrial fibrillation on apixaban, hypertension and a cardiac stent. A 4 mm punch biopsy of the left forearm revealed superficial dermal fibrosis with mild pseudoepitheliomatous hyperplasia and granulomatous inflammation with scattered multinucleate histiocytes. There were giant cells with dark brown, somewhat round, yeast-like structures, some with internal septation exhibiting moderate staining for PAS, compatible with Medlar bodies suggestive of chromoblastomycosis. The patient was on rosuvastatin, rendering itraconazole not a possible treatment option, and instead the patient underwent curettage and cautery with two bouts of cryotherapy freeze and thaw cycles. A twelve-month follow-up noted a crusted area on the distal aspect of the scar. A shave biopsy of this area revealed pigmented organisms suggesting a recurrence of chromoblastomycosis. A further excisional biopsy was performed, with no evidence of chromoblastomycosis. This case highlights multiple surgical options for the management of chromoblastomycosis in patients where medical management is contraindicated. It highlights the therapeutic challenge of this disease due to frequent recurrence of lesions and that repeat biopsy may be efficacious in monitoring for recurrence.

## Simple Summary

Chromoblastomycosis is prevalent in tropical and subtropical regions such as Australia. It should be considered in patients presenting with chronic verrucous, plaque, or ulcerated skin lesions unresponsive to conventional therapies. Typically, treatment is combination therapy or oral antifungal agents for a prolonged period. It has a high recurrence rate. Presentation is typically in the lower limbs, unlike the case presented here. This case highlights the importance of multiple surgical options in patients who have contraindications to oral antifungals, including both surgical excision and cryotherapy. It also highlights the importance of follow-up visits after treatment cessation to ensure lesion clearance and no recurrence.

## 1. Introduction

Chromoblastomycosis is a chronic, granulomatous, suppurative mycosis of the skin and subcutaneous tissue. It is classified as a neglected tropical disease by the World Health Organisation [1,2]. It is typically caused by traumatic inoculation of dematiaceous fungi of the family Herpotrichiellaceae, commonly the Fonsecaea species [1,3]. It is often present in soil, plants, and decomposing wood and predominately occurs in tropical and subtropical regions [1,2,3,4]. Chromoblastomycosis lesions are often difficult to diagnose due to their polymorphous appearance and can result in episodes of secondary bacterial infections [1,5]. It remains relatively rare in Australia. The immune response in chromoblastomycosis is hypothesised to be a combination of both cellular and humoral immune response, with verrucous lesions having high levels of interleukin 4 and interleukin 10, representing a T-helper 2 pathway, while the more atrophic forms have granulomas with more epithelioid and Langerhans cells and tumour necrosis factor alpha, representing a T-helper 1 response [4,6,7,8,9,10]. It often manifests clinically as asymptomatic lesions, which often leads to delayed presentation to medical care [6,7]. Various clinical classifications have been described, including nodular, verrucous, plaque, tumoral and atrophic [4,9,10]. Most lesions are located on the lower limbs [4,5,6]. In tissues, the fungi display morphology of round/oval thick-walled cells that multiply by septation in two distinct planes, called Medlar’s bodies, representing an invasive form [2,3,4,5,6]. Differential diagnoses are numerous and include cutaneous tuberculosis, botryomycosis, tertiary syphilis, neoplasms, sporotrichosis and phaeohyphomycocis [4,5,6,7,8,9]. Histology is characterised by an epidermis with hyper-parakeratosis, pseudoepitheliomatous hyperplasia and potential microabscesses. The dermis often has dense granulomatous inflammation, and characteristic Medlar bodies may be present [4,5,6,7,8,9]. Fungal culture using Sabouraud agar may be used to isolate and identify species [4,5,6,7,8,9]. It is difficult to treat and is often associated with low cure rates and high relapse rates. Treatment typically consists of long periods of antifungal drugs, often combined with physical treatments like surgery, cryotherapy and thermotherapy [10,11,12].

## 2. Case Report

**Figure 1:**
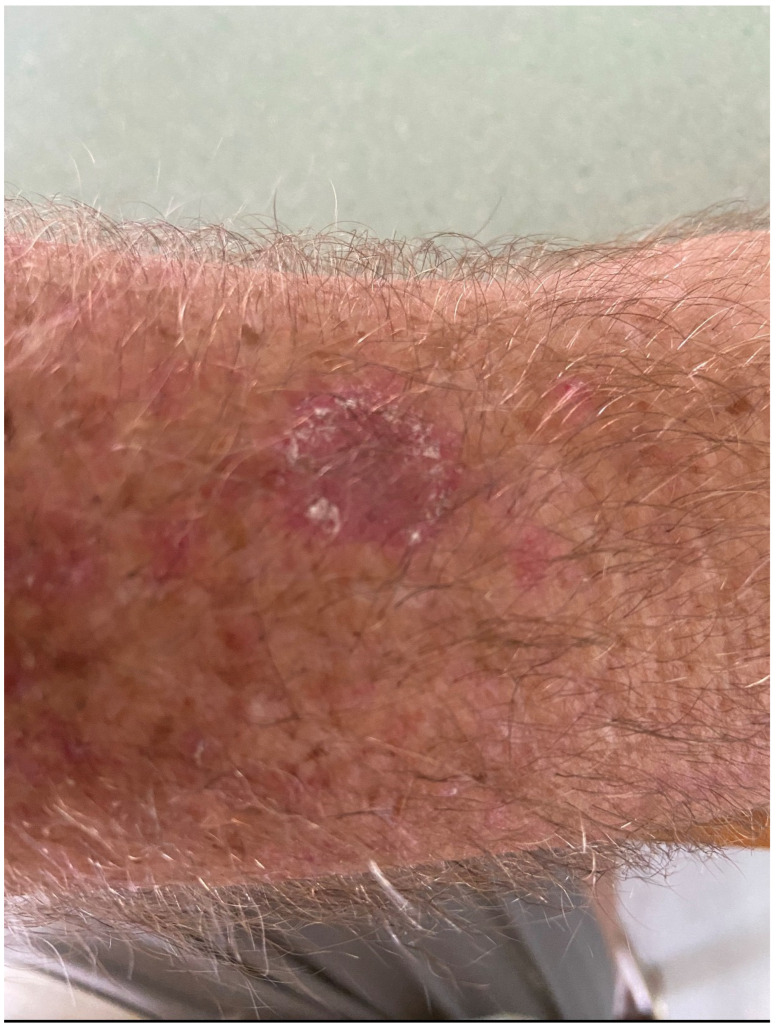
Initial presenting lesion—scaly plaque on the distal forearm.

**Figure 2:**
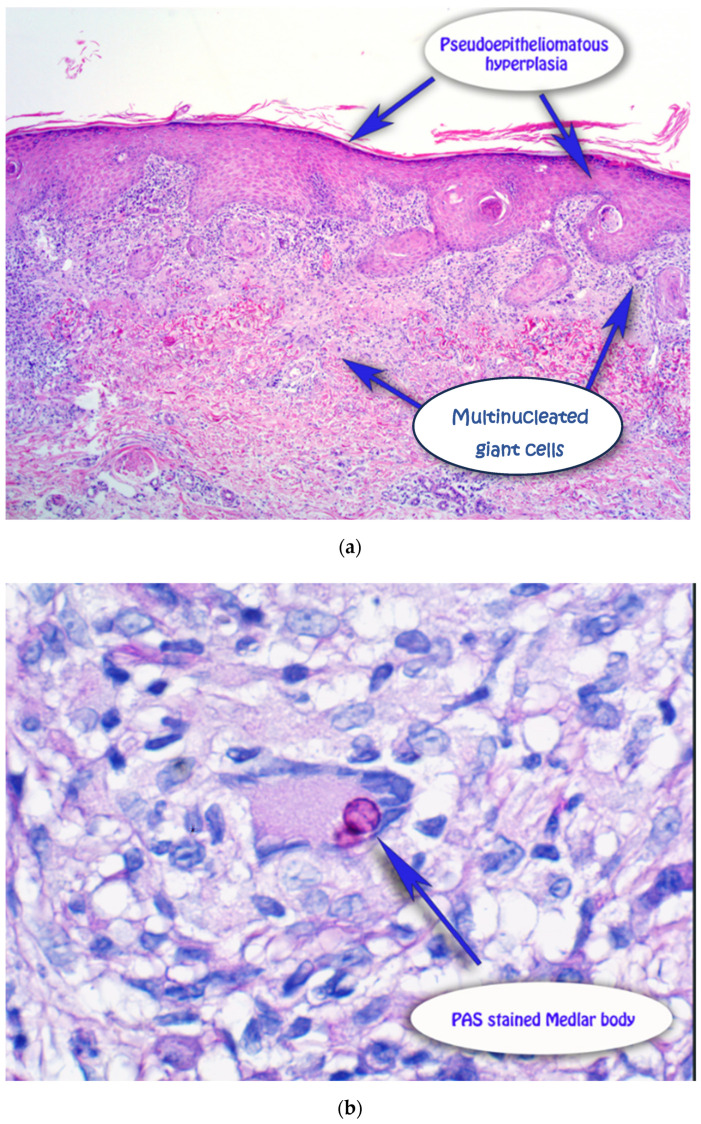
(**a**): H + E of 4 mm punch biopsy demonstrating pseudoepitheliomatous hyperplasia and multinucleated giant cells. (**b**) H + E of 4 mm punch biopsy demonstrating pseudoepitheliomatous hyperplasia and Periodic acid–Schiff-stained Medlar bodies.

**Figure 3:**
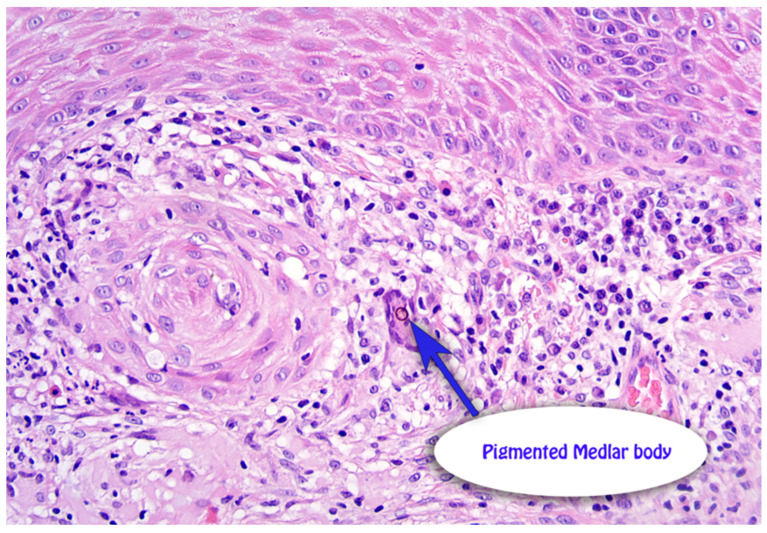
Twelve-month follow-up H + E demonstrating ongoing evidence of Medlar bodies.

A 59-year-old male presented with a 12-month history of an asymmetrical, scaly plaque on the left forearm that was increasing in size (Figure 1). The lesion was not pruritic or painful and had no obvious preceding trauma. The patient was a retired print worker who enjoyed gardening. His past medical history included atrial fibrillation on apixaban, hypertension on rosuvastatin, anxiety/depression on fluoxetine and a recent cardiac stent. A 4 mm punch biopsy was taken from the left forearm. The punch demonstrated superficial dermal fibrosis with mild pseudoepitheliomatous hyperplasia and granulomatous inflammation with scattered multinucleate histiocytes (Figure 2a). Giant cells with dark brown, somewhat round, yeast-like structures, some with internal septation, exhibiting moderate staining for PAS, compatible with Medlar bodies, which were suggestive of chromoblastomycosis (Figure 2b). The patient was on rosuvastatin and apixaban and was not keen to halt either medication for systemic therapy after a recent cardiac stent, rendering itraconazole not a possible treatment option due to drug-to-drug interactions, specifically with apixaban. Terbinafine could have been a second-line option and potentially viable; however, the patient was on fluoxetine and did not feel comfortable with drug-to-drug interactions with these medications. Given these limitations, the patient instead underwent curettage and cautery with two bouts cryotherapy freeze and thaw cycles as treatment. Patient preference was for the most minimally invasive management with regular follow-up. The initial 6-month review revealed no evidence of recurrence, but the 12 month follow-up noted a crusted area on the distal aspect of the scar. A shave biopsy revealed a squamoproliferative lesion with pigmented organisms suggestive of a recurrence of chromoblastomycosis (Figure 3). A further excisional biopsy was performed and was negative for further chromoblastomycosis.

## 3. Discussion

Chromoblastomycosis is a chronic infection caused by dematiaceous fungi endemic to tropical and subtropical areas [2,3]. Once inoculated into the skin, it is a progressive disease, with the melanised fungi invading cutaneous tissues and resulting in slow-growing lesions [2,3]. The clinical course is often chronic, with continued growth for many years. The initial lesion may be asymptomatic or just a mildly pruritic red to violaceous papule. The differentials for early-stage lesions include foreign body reaction, atypical infections or squamous cell carcinoma [4,5]. In persistent infection, malignant transformation to squamous cell carcinoma has been observed, with many case reports documenting this malignant transformation to squamous cell carcinoma [13]. It commonly occurs in immunocompetent hosts, but in a review by Shenoy et al., 41% of patients with chromoblastomycosis had systemic diseases like diabetes and ischemic heart disease, which underscores the association between compromised immunity and infection risk [2]. It typically occurs in the lower limbs, but rare reported presentations include the face and upper limbs [11]. It occurs more frequently in men than women. Long-standing chronic lesions may be complicated by secondary bacterial infections, lymphoedema and malignant transformation. It can have serious irreversible complications such as disabling cutaneous fibrosis [2,3]. Diagnosis is challenging and usually made by histopathologic identification of clusters of thick-walled cells in cutaneous tissues, such as medlar bodies, muriform bodies, or sclerotic bodies [2,3]. Other histopathological features include dermal fibrosis, increased dermal capillaries and pseudoepitheliomatous hyperplasia [2,3]. If needed, fungal cultures may also be used to identify the causative organism [4,6]. Scrapings for microscopic examination using 10% KOH should be taken from sites where black dots are seen on the surface of the lesion. These represent the transdermal elimination of fungal agents [12].

The treatment of this disease remains a global challenge [10,11,12,14]. Chromoblastomyces pathogens form sclerotic corpuscles in tissue, which often cause hypertrophic scars or fibrosis, making it difficult for topical drugs to penetrate [2,9,10,11,12,14]. This disease has no possibility of healing spontaneously, with the condition having a recurrence rate of more than 40% reported [2,3,6]. Oral antifungal therapy is the mainstay of treatment for chromoblastomycosis. Long-term itraconazole therapy at doses of 200–400 milligrammes daily, generally given for six to twelve months depending on clinical severity, is first-line therapy. Pulsed dosing of itraconazole (400 milligrammes per day for seven days per month) has also been reported to be effective and may offer better cost efficiency with improved compliance [2,3,6]. Terbinafine is a second-line treatment agent, and posaconazole at doses of 800 milligrammes daily may be considered for refractory cases. Locally destructive techniques can be used in conjunction with systemic antifungals. In this patient, these systemic options were not feasible due to patient preference and drug-to-drug interactions [9,10,11,12,14]. Cryosurgery via the application of liquid nitrogen to the affected skin may also be used in conjunction with antifungals in limited disease. Treatment with cryotherapy shows minimal adverse effects, but freezing time and depth have still not been standardised. Curettage is typically not recommended, since it can result in involvement of the lymphatic chain [2,9,10,11,12,14].

Surgical excision may also be considered for localised disease or lesions refractory to antifungal therapy. Wide local surgical excision, as well as Mohs micrographic surgery, have been reported to be successful in the proper clinical context. However, cure rates with this regimen are unsatisfactory (~20–70%), and relapses are common [9]. In this case, the patient initially underwent cryotherapy and curettage due to a contraindication to oral therapy. However, the lesion was refractory to this initial surgical management and required wide local excision. A few case reports have looked at alternative therapy, including imiquimod, a potent TLR7 agonist which stimulates a Th-1-weighted cellular immune response, and acitretin, a systemic retinoid that can be beneficial in the treatment of chronic fungal infections [9,10,11,12]. These agents could provide additional therapeutic options, especially in cases resistant to conventional antifungal therapies. Carbon dioxide fractional laser treatment can increase drug penetration. Regardless of the treatment, long-term monitoring is essential to prevent recurrence, and repeat biopsy may be efficacious in monitoring for recurrence [2,3].

## 4. Conclusions

Overall, this case highlights the therapeutic challenge of this disease, not only due to frequent recurrence of lesions but also due to polypharmacy present in the population. It emphasises the importance of individualised treatment planning in patients with chromoblastomycosis, especially those who have contraindications to systemic therapy. It also highlights the role of vigilant long-term follow-up given the high recurrence rates, even after a 12-month period of disease remission.

## Data Availability

Data sharing is not applicable to this article as no new data were created or analysed in this study.
